# SANS from Salt-Free Aqueous Solutions of Hydrophilic and Highly Charged Star-Branched Polyelectrolytes 

**DOI:** 10.3390/polym8060228

**Published:** 2016-06-08

**Authors:** François Boué, Jérôme Combet, Bruno Demé, Martine Heinrich, Jean-Georges Zilliox, Michel Rawiso

**Affiliations:** 1 Laboratoire Léon Brillouin (CEA-CNRS), CE Saclay, 91191 Gif-sur-Yvette Cedex, France; 2 Génie Microbiologique et Procédés Alimentaires, INRA-AgroParisTech-UPSay, 1 rue Lucien Brétignières, 78850 Thiverval-Grignon, France; 3 Institut Charles Sadron (CNRS-UdS), 23 rue du Loess, BP 84047, 67034 Strasbourg Cedex 2, France; jerome.combet@ics-cnrs.unistra.fr (J.C.); mheinrich@unistra.fr (M.H.); michel.rawiso@ics-cnrs.unistra.fr (M.R.); 4 Institut Laue Langevin, BP 156, 38042 Grenoble Cedex 9, France; bruno.deme@ill.eu; 5 Institut de Chimie (UdS), 1 rue Blaise Pascal, BP 296R8, 67008 Strasbourg Cedex, France

**Keywords:** electrostatics, polyelectrolytes, star polymers, Small Angle Neutron Scattering, scattering peak, Debye length, scaling laws, counterions, condensation, semi-dilute, interpenetration, overlap concentration

## Abstract

Scattering functions of sodium sulfonated polystyrene (NaPSS) star-branched polyelectrolytes with high sulfonation degrees were measured from their salt-free aqueous solutions, using the Small Angle Neutron Scattering (SANS) technique. Whatever the concentration *c*, they display two maxima. The first, of abscissa *q*_1_*, is related to a position order between star cores and scales as *q*_1_* ∝ *c*^1/3^. The second, of abscissa *q*_2_*, is also observed in the scattering function of a semi-dilute solution of NaPSS linear polyelectrolytes. In the dilute regime (*c* < c*, non-overlapping stars), peak abscissa does not depend on concentration *c* and is just an intramolecular characteristic associated with the electrostatic repulsion between arms of the same star. In the semi-dilute regime, due to the star interpenetration, the scattering function – through the peak position, reflects repulsion between arms of the same star or of different stars. The *c* threshold between these distinct *c*-dependencies of *q*_2_* in the dilute and semi-dilute regimes is estimated as c*. Just as simple is the measurement of the geometrical radius *R* of the star obtained from the *q*_1_* value at c* through the relation 2*R* = 2π/*q*_1_*. By considering NaPSS stars of the same functionality with different degrees of polymerization per arm *N*_a_, we find *R* scaling linearly with *N*_a_, suggesting an elongated average conformation of the arms. This is in agreement with theoretical predictions and simulations. Meanwhile the value of *q*_2_* measured in the dilute regime does not allow any inhomogeneous counterion distribution inside the stars to be revealed.

## 1. Introduction

Star-branched Polyelectrolytes (PEs) combine remarkable properties and interesting fundamental issues. Small angle radiation scattering yields a surprisingly direct understanding of the latter.

Applications are ones of compact polymeric objects and, in particular, result from a low viscosity and a strong interaction with multivalent ions. In this respect they can be, on the one hand, assimilated or compared with neutral star-branched polymers and copolymer micelles. On the other hand, they can be compared to linear PEs. The branched architecture modifies linear PE properties such as it does for linear neutral polymers [1,2]. We can quote an expectable different counterion distribution, a distinct dependence upon salt addition, leading to the de-swelling of the PE stars, and finally, their capacity to trap ions larger than linear PEs.

From a fundamental point of view, and more precisely a structural one, again both comparisons can be considered. With respect to the mutual repulsive interaction observed with neutral star-branched polymers in solution of a good solvent [2,3], our vision can be enriched by the PE specificities, such as the existence of additional long-range repulsive forces leading to a related different response to the variation of concentration and a screening effect through the presence of added salt.

However, even richer is the comparison with linear PE solutions. We know that the structure of the latter is very complex, due to the numerous regulation mechanisms of electrostatic charge distributions and potentials and their combination with the high versatility of linear chain arrangements. Although the chain architecture is more advanced, the average structure of the star-branched PE solutions is not increasingly complex. On the contrary, it becomes simpler, mainly by the bias of the existence of separate contributions that intervene in a hierarchy of correlations. Beyond the ones between counterions and arms, there are the ones between arms either belonging to the same star (intramolecular correlations) or the ones belonging to two distinct stars (intermolecular correlations). Moreover, the analysis of the star dispersion state is relatively simple since we can distinguish the dilute regime, where stars can be considered as separated centrosymmetric particles, from the semi-dilute one, where they interpenetrate each other to some extent leading to a composite structure [4,5,6]. In both regimes, the intra- and intermolecular correlations behave differently and give access to simple conformational information. This paper aims at showing how geometrical parameters characterizing the average conformation of macro-ions can be measured more easily and how further knowledge can be gained from star-branched PE, through Small Angle Scattering (SAS).

Several star-like PE systems have been studied by SAS in the last decade. M. Heinrich *et al.* synthesized and studied NaPSS stars with a well-defined small core of crosslinked poly(divinylbenzene), similar to the ones studied here [6]. They could separate intra- and intermolecular correlations using Small Angle Neutron Scattering (SANS) [7]. We will discuss their results below. Systems obtained through micellization in water of poly(tert-butylstyrene)-*block*-poly(sodium styrene sulfonate) (PtBS-*b*-NaPSS) and poly(ethylene-*alt*-propylene)-*block*-poly(sodium styrene sulfonate) (PEP-*b*-NaPSS) copolymers, with the hydrophobic block forming the core and the PE-block forming the arms, were studied by P. Guenoun *et al.* by SANS [8,9]. They found a nearly perfect rod-like stretching (with a characteristic *q*^−1^ scattering behavior) for the PSS arms and proposed picturing the micelle as an urchin. This was supported by remarkable Monte-Carlo simulations [10], which can be compared with those of Molecular Dynamics [11,12,13] as well as scaling theory [14]. Later, in SANS experiments from polystyrene-poly(acrylic acid) PS-PA micelles, W. Groenewegen *et al.* measured separately the PS and PA partial correlation functions [15]. From the radius obtained for the PA-shell, they could study the extension of the PA sequence according to the PA degree of ionization. In all these cases in which the star architecture is obtained through a micellization, the aggregation number and the radius of the core of the micelle have to be considered as additional parameters. On the opposite side, NaPA and CsPA stars synthesized and studied by SAXS a few years after by D. Moinard *et al.* had a negligible core since they were made of four arms [16]. As a result, they mostly showed a scattering behavior relatively close to that of linear PEs.

In reference [6], the number of arms was substantially larger (with an average functionality <*f*> = 12), and the core could still be considered as negligible in size. For salt-free aqueous solutions, whatever the concentration, the total scattering function measured by SAXS displayed two maxima. The first, at *q*_1_*, is related to a position order between star cores. It could be interpreted in terms of a privileged distance due to electrostatic repulsion, or close packing above the critical overlap concentration, c*. The second, at higher *q* value, *q*_2_*, is similar to the broad halo observed in the scattering pattern of a semi-dilute salt-free aqueous solution of linear [17,18,19,20,21] associated with a correlation hole of electrostatic origin [22,23,24]. The distinct concentration dependence of *q*_2_* in the dilute, where it stays constant, and in the semi-dilute regimes, where it increases with *c* due to the interpenetration between arms of all vicinal stars, provides a way to estimate the critical overlap concentration c*. This simple and direct picture was however complicated by the fact that in reference [6], the arms of the NaPSS stars were not completely sulfonated (sulfonation degrees in the range 0.63 < τ_S_ < 0.78). As a consequence PSS arms were actually copolymers, poly(styrene-*co*-styrene sulfonate) that have an amphiphilic character which modifies the q* values in linear chain solutions [25,26,27,28]. This is due to the fact that they tend to adopt a so-called pearl necklace conformation, *i.e.*, a combination of string-like and pearl-like parts all along the chemical sequence, of sizes depending on τ_S_, as predicted by theory [29,30] and confirmed by scattering and simulations [25,26,27,28,31]. Incomplete sulfonation of stars made some conclusions [6] less refined as they could have been in a pure hydrophilic PE case, in particular about the arm length and the effect of counterion spatial distribution.

In the present paper, we consider the same type of stars, but with PSS arms fully sulfonated (τ_s_ close to 100%). Though this may appear only a slight improvement, we show that it is more than that: we have now ideal model systems. They enable small angle scattering experiments quite simple to interpret via very well defined geometric information, to yield a solid ground to test a theoretical description, which would then be a support for understanding more complex systems found in applications.

## 2. Experimental

### 2.1. Materials

Polystyrene (PS) stars were prepared according to a so-called arm first method. PS arms, with a narrow molecular weight distribution, were synthesized by anionic polymerization and, in monocarbanionic chain form, initiate the polymerization of a small amount of divinylbenzene (DVB). Star-shaped PS with a minute core of cross-linked poly-(DVB) is obtained [32]. The smallest possible DVB/(PS living end) ratio is chosen in order to restrict the size of the star core while a reasonable functionality, *f*, is reached. The DVB content of the star τ_DVB_ is defined as the ratio of the number of DVB monomers to the total number of monomers. To remove residual PS linear chains and reduce the width of the *f*-distribution, some PS stars were fractionated by further size exclusion chromatography (SEC). The same technique was also applied to obtain the molar mass distributions of both arms and stars, respectively. Such coupled measurements lead to mean functionalities <*f*> and variances σ^2^ of *f*-distributions [7,33].

PS stars were then sulfonated according to the H.S. Makowski *et al.* procedure [34,35]. After neutralization with sodium hydroxide (NaOH), NaPSS stars were purified by extended dialysis against pure water (conductivity of the order of 1 μS·m^-1^) followed by freeze-drying. Their characterization was carried out by elemental analysis. In this way, the degree of sulfonation τ_s_, defined as the ratio of the number of sulfonated units to the total number of units (DVB core excluded), and the weight fraction of water content τ_w,_ were determined for each NaPSS star. In addition, static light scattering experiments were performed on NaPSS stars in dilute aqueous solutions with added salt (NaCl) in order to verify that the sulfonation process did not disturb the star shape of the initial PS samples. A PS star is referred to as S*x*, in order to identify a star (S) architecture and a rough estimate of the arm degree of polymerization (*x* ≈ <*N*_a_>_N_). For the related NaPSS star, −S*y* is added to the initial PS star reference in order to specify the sulfonated character (S) and the arm degree of sulfonation (*y* = τ_s_ (%)). Table 1 lists the characteristics of the different samples used in this study. We note that the high degrees of sulfonation used in this study (τ_s_ = 91% and 94%) were achieved through a second sulfonation according to the same procedure.

PE solutions were prepared by dissolving NaPSS stars in D_2_O (Eurisotop CE-Saclay 99.9% D) at 50 °C for 1 h, then let stand for at least two days prior to the measurements. Concentrations are then evaluated from measured solute and solvent masses, taking into account the weight fraction of water content of dried NaPSS stars and using the tabulated partial molar volumes [7,36,37].

### 2.2. SANS Measurements

SANS experiments were conducted on both diffractometers: PACE at LLB, CEA-Saclay, Gif-sur-Yvette, France; and D22, at ILL, Grenoble, France. Measurements on PACE were done using three distinct configurations (sample-to-detector distances *D* = 1, 3, and 4.6 m with wavelengths *λ* = 6, 10, and 13 Å, respectively) covering the *q*-range 0.003 < *q* < 0.322 Å^−1^. Those on D22 used two configurations (*D* = 1.5 and 15 m with λ = 6 Å) leading to the *q*-range 0.004 < *q* < 0.544 Å^−1^. *q* is the magnitude of the scattering vector, defined by the wavelength of the incident beam *λ* and the angle between incident and scattered beams θ such that *q* = (4π/λ)·sin(θ/2). The scattered intensity was recorded by: a multidetector with 30 concentric rings of 1 cm width, on PACE; a 2-dimensional ^3^He gas detector, on D22. PE solutions were held in circular and calibrated quartz cells of distinct thickness 1, 2.5, or 5 mm according to their concentration. Multiple scattering was then negligible. Also, the cells were mounted in a temperature-controlled sample holder. Most of the measurements were done at 20 °C, but a few others at 25 and 65 °C.

The scattering data were treated according to the usual procedures for isotropic SANS [38,39,40]. They were first corrected for electronic noise of the detector, empty cell, sample transmission, and sample thickness. Geometrical factors and detector cells efficiency were then accounted for, using the (flat) incoherent scattering of H_2_O. Finally, the normalization to the unit incident flux was carried out through either the direct measurement of the number of incident neutrons (PACE) or the scattering of H_2_O 1mm thick (D22). In this way, the differential cross section per unit volume ∑(*q*) of each solution is obtained in absolute units (cm^−1^). It is the sum of a coherent cross section term containing all the information needed to describe the structure of the solution, *I*(*q*), and a background term, ∑^B^, which has to be removed from ∑(*q*) to obtain *I*(*q*). ∑^B^ involves the scattering of the solvent and the incoherent scattering of the macromolecules (macro-ion plus counterions for a PE). For PE solutions, it can be readily replaced by the scattered intensity of a specific mixture of D_2_O and H_2_O solvent molecules. The D_2_O volume fraction of the equivalent D_2_O-H_2_O mixture is then assessed via a method described in references [7,27,28,38].

At this stage, to make meaningful the comparisons between distinct polymers and distinct solutions of the same polymer, we consider the dimensionless scattering function *g*(*q*,*c*) defined as:

*g*(*q*,*c*) = *S*(*q*,*c*)/*cN_Av_* = *I*(*q*,*c*)/<*K*>^2^*cN_Av_* (1)

*I*(*q*,*c*) (cm^−1^) corresponds to the quantity *I*(*q*) defined above when measured at a given concentration *c*, <*K*>^2^ (cm^2^) to the squared contrast factor between the polymer unit and the solvent (see Equation (2) below); *S*(*q*,*c*) (cm^−3^), to the scattering function of the polymer per unit volume; *c* (mol·cm^−3^), to the polymer concentration of the solution (mole of repeating unit par unit volume); *N_Av_* (mol^−1^), to Avogadro’s number.

For solutions of parent PS stars in THF, the average contrast length <*K*> is obtained from the relation:

<*K*> = τ_DVB_ · *K*_DVB_ + (1 − τ_DVB_) · *K*_PS_ (2)

*K*_DVB_ and *K*_PS_ (cm) are the “contrast lengths” of the elementary scatterers DVB (C_10_H_10_) and PS unit (C_8_H_8_) in deuterated THF, respectively. For aqueous solutions of NaPSS stars, <*K*> can be estimated from the relation:

<*K*> = τ_DVB_ · *K*_DVB_ + (1 − τ_DVB_) · τ_S_ . *K*_PSS_^−^ + (1 − τ_DVB_) · (1 − τ_S_) · *K*_PS_ (3)

*K*_DVB_, *K*_PSS_^−^ and *K*_PS_ are now the contrast lengths of the elementary scatterers DVB (C_10_H_10_), PSS^−^ repeat unit (C_8_H_7_SO_3_^−^) and PS repeat unit (C_8_H_8_) in D_2_O, respectively. These contrast lengths are computed from the coherent scattering lengths and partial molar volumes listed in the literature [7,36,37,41]. They depend on temperature via the related changes in the solvent molar volume. Indeed, the dilatation coefficients for the volume of both kinds of unit are much lower than the ones of simple liquids [42]. So, only the T-dependence of the D_2_O molar volume must be taken into account in the calculation of the contrast lengths. The fraction of H_2_O content of dried NaPSS stars was also accounted for. It leads to an additional slight *c*-dependence of the contrast lengths.

Such data normalization is only approximate for NaPSS aqueous solutions, since we have a multicomponent solute made of large macro-ions and small counterions. The use of Equations (1) and (3) amounts to neglecting counterions and assuming that *g*(*q*,*c*) and *S*(*q*,*c*) in Equation (1) correspond to the partial scattering functions associated with the repeating units of the macro-ions, not the counterions. This is justified because we are concerned with SANS. The related contrast factor of Na^+^ counterions in D_2_O is indeed much lower than the one of PSS^−^ units in D_2_O. We will not detail here how that actually results from the hydrophilic character of Na^+^ counterions and the so-called electrostriction effect (negative partial volume) [7,36,37]. We just keep in mind this advantage of SANS for PE solutions: it allows measuring of the scattering function of the macro-ions (the chains), provided the counterions are sufficiently hydrophilic. The situation is different for SAXS where the counterion contribution to the scattering signal cannot usually be neglected [6]. As shown elsewhere [43], this leads to some ambiguous and therefore undesirable shift in the position q* of the “PE peak” associated with linear PE solutions. As a consequence, the power law q* ∝ *c*^1/2^, predicted for the semi-dilute solutions of linear and hydrophilic PE within the isotropic model, is observed in a large concentration range only if the macro-ion partial scattering function is measured. If the counterion contribution to the scattering signal is no longer negligible, the *c*^1/2^ scaling law is no longer perfectly obeyed. This is another difference and advantage of the present study, which uses SANS with respect to that reported in Ref. [6] that used SAXS.

## 3. Results and Discussion

### 3.1. Results: Effects of Concentration c and Arm Degree of Polymerization N_a_

**Figure 1:**
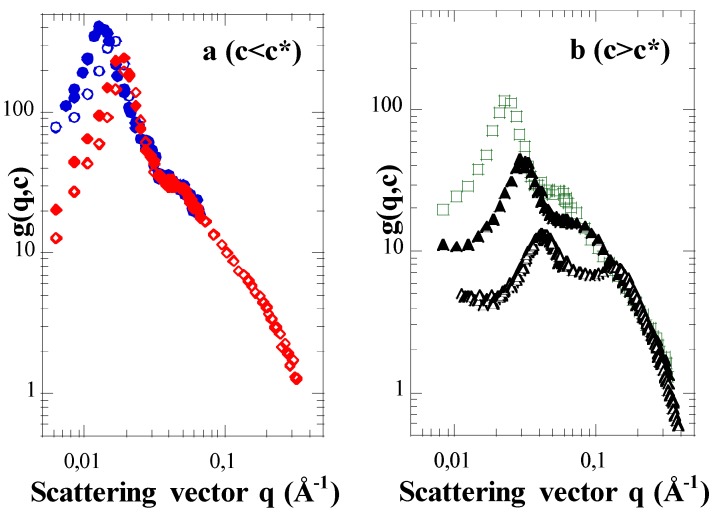
Scattering functions of NaPSS star solutions in the dilute and semi-dilute regimes. (**a**) S100-S91/D_2_O in the dilute regime (*c* < c* ≈ 0.075 mol·L^−1^ – *φ* < φ* ≈ 0.83%); *c* = 0.013 mol·L^−1^ – *φ* = 0.14% (dotted circles); *c* = 0.025 mol·L^−1^ – *φ* = 0.28% (circles); *c* = 0.038 mol·L^−1^ – *φ* = 0.42% (dotted lozenges); *c* = 0.052 mol·L^−1^ − *φ* = 0.58% (lozenges); (**b**) S100-S91/D_2_O in the semi-dilute regime (*c* > c*); *c* = 0.102 mol·L^−1^ − *φ* = 1.13% (squares); *c* = 0.258 mol·L^−1^ − *φ* = 2.87% (dotted triangles); *c* = 0.754 mol·L^−1^ − *φ* = 8.39% (triangles).

**Figure 2:**
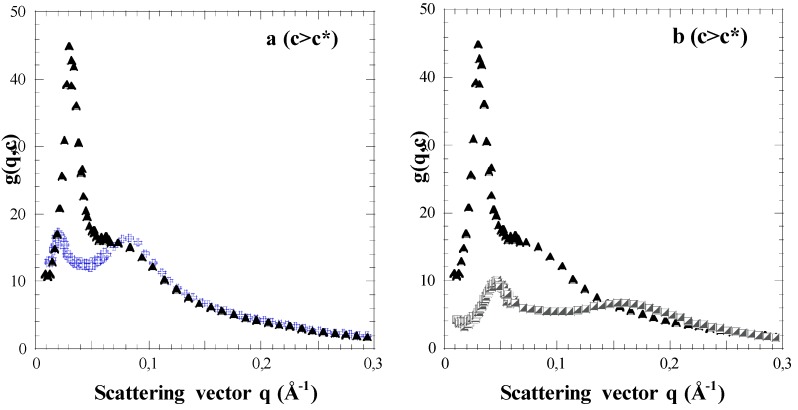
Scattering functions of NaPSS star solutions in the semi-dilute regime. (**a**) Changes according to *N*_a_; S100-S91/D_2_O *c* = 0.258 mol·L^−1^ − *φ* = 2.87% (dotted triangles); S300-S94/D_2_O *c* = 0.230 mol·L^−1^ – *φ* = 2.57% (crosses); (**b**) Changes according to *c*; S100-S91/D_2_O *c* = 0.258 mol·L^−1^ – *φ* = 2.87% (dotted triangles); S100-S91/D_2_O *c* = 0.996 mol·L^−1^ – *φ* = 11.08% (half-dotted squares).

The scattering functions of the parent PS stars (non sulfonated, organic solvent) are not shown in this paper (see Ref. [6]). We mainly focus on the scattering functions *g*(*q*,*c*), or *g*(*q*,*φ*) of the related fully sulfonated NaPSS stars. Their evolutions with the unit concentration *c*, or the corresponding volume fraction *φ*, as well as the number average arm degree of polymerization <*N*_a_>_N_ (noted *N*_a_ hereafter) are shown in Figure 1 and Figure 2.

Whatever the concentration, we observe at least two maxima, even though the second maximum is rather like a shoulder in the dilute regime. This is at variance with both neutral PS stars and linear NaPSS polyelectrolytes, for which only one maximum is observed. Moreover, the positions of these two successive maxima in the reciprocal space, *q*_1_* and *q*_2_* with *q*_1_* < *q*_2_*, vary differently with concentration. In a region of low *c* (Figure 1a), (identified as the dilute regime (*c* < c*)), *q*_1_* varies with *c*, while *q*_2_* remains constant. Conversely, at high *c* (Figure 1b), namely in a concentration range *c* > c* corresponding to the semi-dilute regime, both maxima are shifted to larger and larger *q* values. The related variations are however distinct. This difference in c-dependence of the two maxima is also clear-cut when the arm degree of polymerization *N*_a_ of the stars that have almost the same functionality is varied (Figure 2a). For the same concentration *c* above the critical overlap concentration c*, the second maximum is at the same position *q*_2_*, for *N*_a_ = 96 and *N*_a_ = 280, while the position as well as the shape of the first maximum varies. Its position, *q*_1_*, decreases while its height lowers as *N*_a_ is increased. The differences in the two curves for *c* constant and two values of *N*_a_ are parent of those at *N*_a_ constant and two concentrations in Figure 2b: the curve for the largest concentration with *N*_a_ = 96 is similar to the one for the largest *N*_a_ = 280 at the lowest *c*. In both cases, the lowering of the height of the low *q* maximum indicates that the concentration fluctuations are reduced and/or more screened: this can be interpreted as due to a dispersion state of higher interpenetration of the stars. We can also emphasize that the amplitude changes of both maxima, in the semi-dilute regime, are correlated. The first one thus disappears for the benefit of the second one as concentration increases, and reciprocally. That suggests some composite structure for *c* > c*, made of two distinct media which overlap each other [4,5,6,7].

### 3.2. First Discussions: Using the Variations of the Maxima Positions q_1_* and q_2_* with Concentration c to Attribute Them to Defined Distances (“Indexation”)

**Figure 3:**
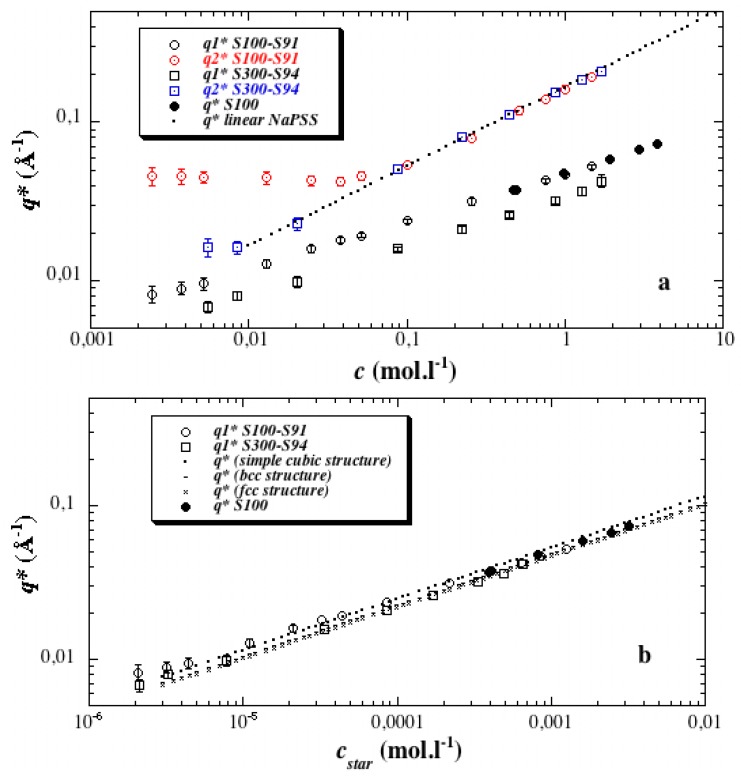
Indexation of the main maxima of the PS and NaPSS star scattering functions. (**a**) Scattering vectors q* (PS) [1], *q*_1_* and *q*_2_* (NaPSS) *versus* statistical unit concentration *c*. The scaling law associated with NaPSS linear polyelectrolytes of sulfonation degree close to one is also plotted (q* = (0.165 ± 0.002)*c*^1/2^ [18,20]); (**b**) q* (PS) [1], *q*_1_* (NaPSS) *versus* star concentration *c*_star_ (not to be confused with c*). The expected scaling laws for a simple cubic lattice (. . .), a body-centered cubic (bcc)-lattice (- - -) and a face-centered cubic (fcc)-lattice (× × ×).

We consider now Figure 3a which contains most of the information enclosed in this paper, through the simple survey of the main features of the c-dependence of the position of each kind of maxima. It allows us to lay stress on the difference in nature of both maxima. It turns out that it enables their indexation. Indeed, both show power laws for q* according to *c* (seen as straight lines in log-log representation), but with different exponents (slopes in log-log representation).

First, *q*_1_* (open circles and open squares) almost scales as *c*^1/3^ on the whole explored concentration range, with different front factors for the two stars S100-S91 and S300-S94, *i.e.*, for the two distinct *N*_a_ values (the two lines are shifted in Figure 3a). This c-dependence is different from the one found for the polyelectrolyte peak of linear NaPSS polyelectrolytes in the semi-dilute regime, which is close to *c*^1/2^ (dotted line in Figure 3a). The exponent 1/3 suggests that the first maximum corresponds to a position order in three dimensions. *q*_1_* is then related to a mean distance between objects in a 3d liquid- or crystalline-like structure (scaling laws for simple cubic, bcc, and fcc lattices are presented in Figure 3b). From this point of view, it is noticeable that scattering functions of parent PS stars in THF, a good solvent, show similar maxima with identical positions q* for the same concentration (Figure 3a; q* for S100, full circles, is identical to *q*_1_* for S100-S91, open circles, at the same concentration). This similarity between neutral and charged stars gives further support to a direct relation between *q*_1_* and the mean distance between star cores, 2π/*q*_1_*. By the way, it also confirms nicely that no significant degradation of the star-branched architecture occurs under the sulfonation process. Finally, all the *q*_1_* values can be roughly superimposed into a single curve if the star concentration *c*_star_ (not to be mistaken with c*) is used as variable instead of the polymer concentration *c* (Figure 3b). Taking into account polydispersity, *c*_star_ is defined as:

*c*_star_ = (<*m*>/*M*_N_)*c* (4)

*M*_N_ is the number average molecular weight of the star; <*m*>, the molar mass of its effective units (Table 1); *c*, the statistical unit concentration.

In the same Figure 3b, we also plotted for comparison in dotted lines the variations of q* for different kinds of compact crystalline structure. The general agreement and accuracy are quite satisfying. We notice the slight departure from the *c*^1/3^ scaling law as the concentration increases. It may be ascribed to the fact that at large concentrations, *q*_1_* enters the *q* region where the star form factor shows a steeper variation, producing a shift to lower *q*. It could also be ascribed to some increase in packing compactness as concentration increases, which would be more sensitive for lower *N*_a_.

As a final remark on the variation of *q*_1_*, we stress the fact that the *c*^1/3^ scaling law shows a continuous variation when the solution passes c*. This classical behavior does not allow us, in principle, to differentiate between interpenetration of the stars and a progressive shrinking (compaction) of each star keeping them at the overlapping concentration. A better insight could come from the analysis of the shape of the scattering maximum, but modeling is beyond the scope of this paper. We discuss below (see Section 3.6) another fact in favor of interpenetration.

Let us now turn towards the second type of maximum observed at *q*_2_* > *q*_1_*. Since this maximum never exists for neutral PS stars, it has an electrostatic origin. Its position dependence upon polymer concentration shows that it is not commensurate with the first one (Figure 3a).

For low *c*, *q*_2_* is found to be constant. Actually, it only depends on the arm degree of polymerization *N*_a_ for a fixed mean functionality <*f*>. For a given *N*_a_, we attribute this to the fact that the average internal polymer concentration *c*_i_ inside each individual star is constant, as long as the stars do not overlap each other. This regime actually corresponds to the dilute regime, *c* < c*.

For *c* above a certain threshold, *q*_2_* starts to increase. Our interpretation is that stars begin to interpenetrate each other; therefore their average inner concentration increases. In practice, this could be related to either an interdigitation or a compaction process. The only knowledge of the peak position (and unfortunately, its limited accuracy around c*) is indeed not enough to distinguish between them since both could in principle lead to a similar variation of the average correlation length with *c*. Since the number of arms is rather limited, we consider hereafter the interdigitation process. Whatever the real situation is, the concentration at which q* passes from a plateau to an increasing line is therefore interpreted as the overlap threshold, c*. Note the interesting fact that in this interpenetrating range *c* > *c* *, the *q*_2_* values are found to be unaffected by the arm degree of polymerization *N*_a_. All *q*_2_* values fall over the same dotted line, which corresponds correctly to the variation q*(Å^−1^) = (0.165 ± 0.002)·*c* (mol·L^−1^)^1/2^ found for linear fully sulfonated NaPSS [18,19,20,21]. Therefore, when the stars studied here (with a point-like center) are interpenetrated, the average correlations behave as if the repeat unit’s concentration was spatially homogeneous inside the star and equal to the nominal one, *c*.

The exponent of the scaling law *q*_2_* ~ *c*^α^, with α = ½, for all these highly sulfonated stars, is at variance with the lower value of α found for NaPSS stars of lower degree of sulfonation 0.63 < τ_S_ < 0.78 in Ref. [6]. The latter variation agreed with the one found from partially sulfonated linear PSS solutions (e.g., q* = 0.130*c*^0.465^, for τ_S_ = 0.69, from SAXS measurements of [25,26]). Thus, we get the pleasing result that the *q*_2_*(*c*) function is the same for interpenetrated stars and for interpenetrated linear chains of equal τ_S_, both in the case of τ_S_ = 0.69, and in the case of τ_S_ close to 1.

Similarly, we note that for a given arm degree of polymerization *N*_a_, the value of the *q*_2_* plateau in the dilute regime is lower for this paper (0.045 Å^−1^ for *N*_a_ = 96 and τ_S_ = 0.91) than for Ref. [1] (0.06 Å^−1^ for *N*_a_ = 96 and τ_S_ = 0.65). This is related to the difference in c*, which is smaller for total sulfonation than for partial sulfonation (0.075 mol·L^−1^ compared to 0.165 mol·L^−1^, with *N*_a_ = 96, for τ_S_ = 0.91 and 0.65, respectively): stars overlap here at lower concentration *c* because their arms are more extended, and this is consistent with a lower concentration inside the star.

All these new observations result from the hydrophilic character of the PSS stars studied in the present paper. Such a character is confirmed by the study of the temperature effect, as we see below.

### 3.3. Effect of Temperature

**Figure 4:**
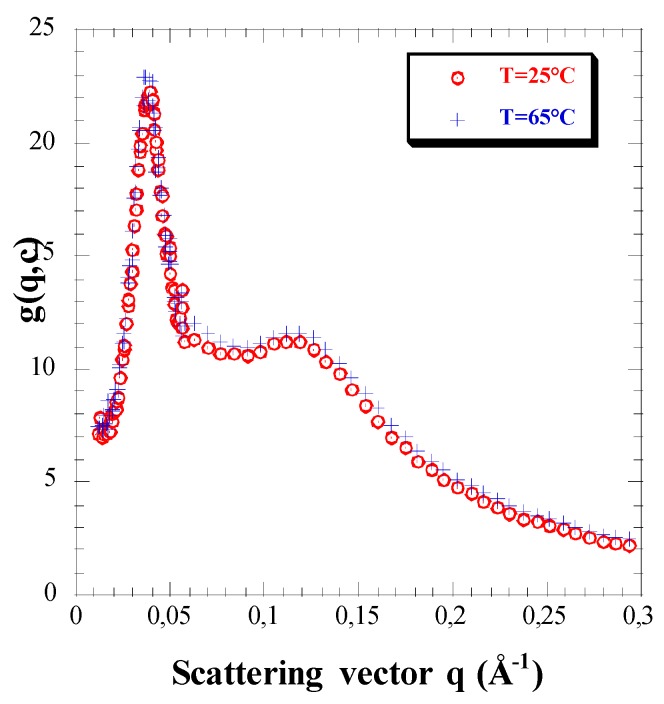
Scattering functions of the same NaPSS star solution (S100-S91 in D_2_O at *c* = 0.480 mol·L^−1^ – *φ* = 5.34%; *c* > c* = 0.075 mol·L^−1^ – φ* = 0.83%) at two distinct temperatures 25 °C (circles) and 65 °C (crosses).

Figure 4 displays the scattering functions obtained from the same solution at two different temperatures 25 and 65 °C. They show definite absence of any temperature effect in this range. We find the same lack of hydrophobicity as checked in completely sulfonated linear PSS [44]. On the one hand, the lack of T-dependence here is strong support for the high degree of sulfonation of our stars, since for lower sulfonation rates a temperature effect has indeed been observed in the same range [7,45]. On the other hand, it can be considered as another test of hydrophilicity of highly sulfonated PSS chains, since if the unit concentration increased when closer to the center of the star, this could make it more sensitive to hydrophobicity, which remains unnoticeable.

### 3.4. Direct Estimate of the Star Size from the c Dependence of the First Maximum and Comparison with Theories

#### 3.4.1. Estimate of the Size *R* from the Star Overlap Concentration

**Figure 5:**
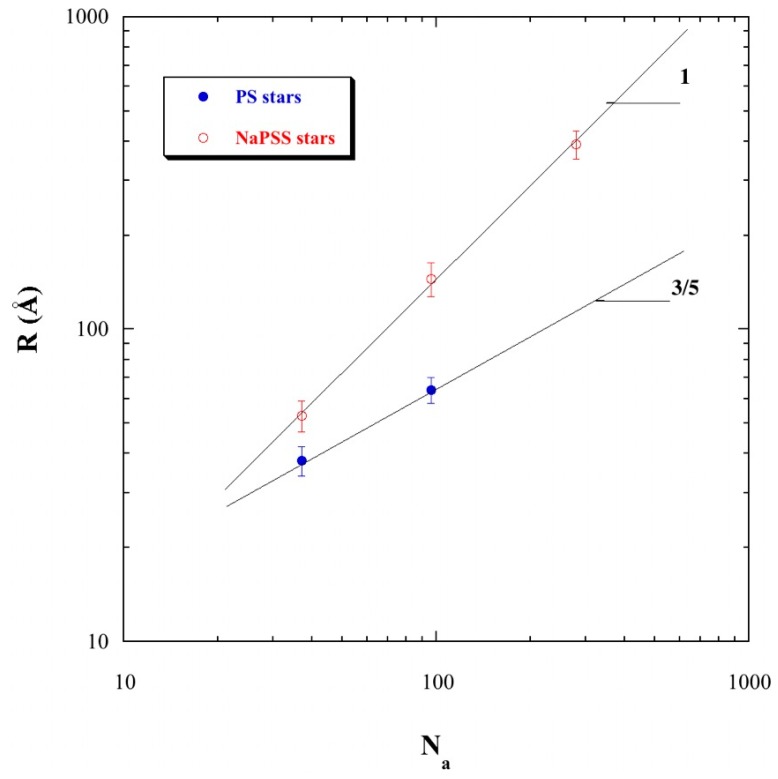
Mean radii of NaPSS stars (*R*) according to their arm degrees of polymerization (*N*_a_). For each star polyelectrolyte, *R* is obtained from the *q*_1_* value at c* (*R* = π/*q*_1_*). A comparison with some precursor PS stars is also offered.

At the critical overlap concentration c*, the stars are packed in contact with each other. Their center-to-center average distance is therefore equal to 2*R*, where *R* is their (geometrical) radius. *R* can also be considered as the mean end-to-end distance of each arm. The precise value of c* is known from the change in the variation of *q*_2_* with *c*. Having checked the different models of packing in Figure 3b, we can use directly the relation *q*_1_* = 2π/2*R* at *c* = c* to determine the radius of the stars. The obtained values of *R* for the three quite different arm degrees of polymerization *N*_a_, 36, 96, and 280 are plotted in Figure 5.

These values of *R* are compared with the ones for the “parent” PS stars, before sulfonation, in good solvent (their related scattering functions can be found in Ref. [5]). In the latter case *R* varies as *N*^3/5^, where 3/5 is approximately the exponent for the self-avoiding random walk, or a chain in good solvent. For PE stars, the three points *R*(*N*_a_) are aligned in log-log plot, with a slope clearly equal to one, suggesting strongly elongated arms. This is in agreement with the linear scaling law (*R* ∝ *N*_a_) predicted by the scaling theories [14,46,47], and with simulations [10,11,12,13]. The front factor of the scaling law here is close to 1.41 Å per repeat unit that indicates a rather extended average conformation for the arms.

#### 3.4.2. Comparing with Scaling Theory

It can be compared first with that of linear PSS macro-ions, which can be calculated from the scaling theory [24]. Indeed, a strongly charged PE can be roughly treated as a weakly charged one with an effective charge fraction *f*_eff_ = *a*/*l*_B_, for monovalent counterions. Here, *a* is the contour length of the monomer unit, which is equal to 2.5 Å for PSS; *l*_B_, the Bjerrum length that is equal to 7.12 Å in water at room temperature (hence *f*_eff_ = 0.35). The end-to-end distance for linear macro-ions in theta solvent is then [24]:

*R* ≈ *N*·*a*·[*f*_eff_^2^ (*l*_B_/*a*)]^1/3^ = *N*·*a*·(*a*/*l*_B_)^1/3^ (5)

With an electrostatic blob size:

*ξ*_el_ ≈ *a*·[*f*_eff_^2^ (*l*_B_/*a*)]^−1/3^ = *a*·(*l*_B_/*a*)^1/3^ (6)

Equations (5) and (6) lead to a scaling law *R*(Å) ≈ 1.76 N and an electrostatic blob size *ξ*_el_ ≈ 3.54 Å for linear PSS macro-ions. Hence our measurement is close (1.41 instead of 1.76) to the prediction of the scaling theory for linear chains in the dilute regime, and therefore, in a very extended state [24]. This prediction is very close to the prediction from the scaling approach of Shusharina and Rubinstein for the dilute osmotic regime of star polyelectrolytes [14]. In this approach, the radius of a star is determined by the balance of (i) the counterion entropy favoring the increase in the star volume and (ii) the entropic elasticity of arms that opposes this expansion. Balance of these two contributions then leads to a star radius independent of the number of arms and close to the end-to-end distance of a linear chain associated with one arm. In other words, the arms of the star are not stretched with respect to the size of a corresponding individual PE chain with counterions condensed on it. In practice we summarize that our experimental result for *R* agrees with both equal scaling predictions, the one for a linear chain [24] and the one for the dilute osmotic regime of stars [14].

#### 3.4.3. Comparison with the Wormlike Chain Model

Beyond the comparison with the scaling approach, it is important to mention that the front factor of the linear scaling law determined for the radius of the NaPSS stars is also similar to the contour lengths per repeat unit—an “effective repeat distance” which we note *a*_eff_, obtained from the analyses of the measured form factors for fully sulfonated PSS chains through the wormlike chain model, estimated between 1.7 and 2.5 Å [48,49,50]. This also means that both for linear chains and stars, the electrostatic blob size is not far from that of the repeat unit.

Moreover, a comparison with a model of chain conformation is important for the description of the arm conformation. The scattering curves reported in this paper do not provide a direct measurement of the form factor. However, the radius extracted as discussed above gives at least a direct measurement of the end-to-end distance of the arms *R*. In the frame of the wormlike chain description, it can be written:

*R*^2^ = 2*l*_p_ × [*L* − *l*_p_(1 − exp(−*L*/*l*_p_))] = 2*l*_p_ × [*Na*_eff_ − *l*_p_(1 − exp(−*N*(*a*_eff_/*l*_p_))] (7)

*L* = *Na*_eff_ is the chain contour length; *N*, the degree of polymerization; *a*_eff_, the effective contour length per repeat unit; *l*_p_, the persistence length. For *l*_p_/*a*_eff_ very large (or very small *L*/*l*_p_ values), Equation (7) reduces to *R* ~ *N*·*a*_eff_. We will see below that in our experiments, for each value of *N*, the obtained value of *l*_p_ is such that the contour length and the radius of the stars only represents a few persistence lengths *l*_p_.

To be more accurate, we consider the Zimm approximation of the Schulz distribution for the degree of polymerization:

*w(N) = (1/k!)·(k/N_N_)^k+1^ ·N^k^·*exp*(-k·N/N_N_)* (8)

where *N*_N_ is the number-average degree of polymerization and *k* is related to the polydispersity index *I* by the relation *I* = *N*_W_/*N*_N_ = (*k* + 1)/*k*.

The number-average of the square of the end-to-end distance is therefore:

(9) $${<R^{2}>}_{N}=2l_{p}^{2}[\frac{a_{\mathrm{eff}}}{l_{p}}N_{N}-1+{\left(\frac{k}{\left(\frac{a_{\mathrm{eff}}}{l_{p}}N_{N}+k\right)}\right)}^{k}]$$

Thus, accounting for the arm polydispersity according to Equation (8) and using <*N*_a_>_N_ (listed in Table 1), as well as a common average effective monomer size *a*_eff_ = 2.1 Å, we can find solutions for *l*_p_: *l*_p_ = 29 Å, for <*N*_a_>_N_ = 36 (*R* = 53 Å); *l*_p_ = 80 Å, for <*N*_a_>_N_ = 96 (*R* = 145 Å); *l*_p_ = 180 Å, for <*N*_a_>_N_ = 280 (*R* = 391 Å). As announced above, within this approach, the radius of the stars *R* and the chain contour length (*L* = *N*_a ·_ *a*_eff_) only involve a few persistence lengths (*R* ≈ 2*l*_p_, and *L*/*l*_p_ ≈ 3). This leads to a linear relationship between *R* and *N*_a_. The observed variation of *l*_p_ with the degree of polymerization can be surprising. However it may simply result from the variation of the average internal concentration in the stars *c*_i_, which depends on *N*_a_ and which can be directly approximated to c* (where stars are in contact and fill the space).

*c*_i_ ≈ c* ∝ <*f*>·*N*_a_/*R*^3^ (10)

If we compare *l*_p_ values with that of linear NaPSS chains (in the semidilute range) for a monomer concentration equal to *c*_i_, we find that the two variations are close (see Figure S1 in Supplementary Materials using data from Ref. [18] and others). The conformation of arms in the stars, and more precisely their persistence length, therefore resembles that of linear chains in semidilute regime at the same average concentration (we note however that the degrees of polymerization of the corresponding linear chains are much larger than *N*_a_).

The observed similarity with linear PE solutions implies that the average ionic strength and the counterions distribution around the chains would be the same in stars as for linear chains. However the star shape, at variance with a linear chain, could in principle lead to an inhomogeneous distribution of the counterions, and therefore of the Debye screening length, inside the stars. This is predicted, by theory and simulations [10,11,12,13,31], for the highly charged PE stars (also called “osmotic stars”). The experimental aspect is detailed in the next paragraph.

### 3.5. Counterions Condensed and/or Trapped Inside the Stars: Information from the Second Maximum

#### 3.5.1. Estimating the Inner Counterions Concentration from *q*_2_*

The second maximum in the scattering functions of PE stars represents the electrostatic repulsion between arms. In the dilute regime, it is exclusively concerned with the repulsion between arms belonging to the same star. Indeed, it is an intramolecular characteristic, as proved by the superimposition of the scattering functions *g*(*q*,*c*) associated with distinct values of *c* < c*, in the corresponding *q*-range (Figure 1a).

It has been often assumed that the correlation length associated to the position of this second maximum *q*_2_* is connected to an average internal screening length *K*_i_^−1^, through the scaling law *q*_2_* ∝ *K*_i_ [6]. *K*_i_^−1^ is an average Debye screening length, which can be linked to the ionic strength and therefore, to an average inner concentration of counterions *c*_ion_ through:

*K*_i_^2^ = 4π*l*_B_*c*_ion_ (11)

Counterions can be condensed around the chains, or simply trapped between the arms. If we consider that condensed ions do not participate to the screening and that only trapped ions (of concentration *c*_trapped_) are active

*K*_i_^2^ = 4π*l*_B_*c*_trapped_ (12)

As *q*_2_* does not depend on concentration in the dilute regime (Figure 3a), we can assume, *c*_ion_ or *c*_trapped_ to be constant for *c* < c*. Equation (12) is also valid in the semidilute regime (*c* > c*) where *q*_2_* varies as *c*^1/2^ (trapped ions can now be viewed as classical “free ions” in a semidilute solution). Since *q*_2_* gradually evolves from a constant value to the *c*^1/2^ regime, we can argue that the concentration of trapped ions in the dilute state coincides with that of the free ions just above c*. This indicates that most counterions are confined inside the stars and show a condensation process similar to that of linear chains, as expected from the scaling approach of Shusharina and Rubinstein for osmotic star PEs [14].

In the dilute state, the fixed concentration of trapped counterions corresponds to a fraction of the internal monomer concentration *c*_i_ and is thus related to the overlap concentration c*. Therefore, Equation (12) becomes:

*K*_i_^2^ ∝ 4π*l*_B_c* ∝ 4π*l*_B_*c*_i_ ∝ <*f*> *N*_a_/*R*^3^ (13)

Thus, as *R* is proportional to *N*_a_, and *q*_2_* *K*_i_, we obtain:

*K*_i_^2^ ∝ *N*_a_^−2^ and *q*_2_* ∝ *N*_a_^−1^ ∝ *R*^−1^, *i.e.*, *q*_2_*R ∝ *q*_2_*N_a_ = *cst* (14)

Both relationships are confirmed by the scattering data obtained with the three NaPSS stars, as indicated in Table 2. Combining Equation (10) with the dependence of *R* over *N*_a_, leads to a scaling law previously established for linear hydrophilic PE, c* ∝ *N*_a_^−2^. The three couples (c*, *N*_a_) associated with our experiments fit the curve previously established from SAXS and viscosity results obtained with NaPSS linear PEs [51], as shown in Figure S2. This is in favor of lengths of the arms similar to that of a linear chain in dilute solution, as predicted by theory.

#### 3.5.2. A Heterogeneous Radial Distribution? Present State of the Art

Until now we reasoned on an average internal counterions concentration. We now return to an important feature for stars, which is the possible existence of an r-dependence of the internal screening length distribution, related to that of the mean distance between arms. For arms strongly elongated, with end-to-end average distance scaling linearly with the arm degree of polymerization, the repeat unit density *c*_i_(*r*) is expected to scale as *f*/*r*^2^ and correlatively *K*_i_^−1^ would scale as *r*/*f*^1/2^ [14,46,47]. The star architecture therefore would force an average growing of the Debye length *K*_i_^−1^ with *r*. That is in agreement with the observation of a change in *q*_2_* according to *N*_a_.

At this stage, we can therefore note in passing a potential difference between PE stars and PE brushes: condensation of the counterions can occur, in a star, closer to the center and let free a large part of the arms from their counterions. P. Guenoun *et al.* [8,9] found extended (*i.e.*, stretched) PSS sequences in copolymer micelles (hence their picture of an urchin), which led to a detailed comparison with simulations [10]. The latter distinguish three different cases according to the coupling parameter *ξ* = *l*_B_/*a* for monovalent counterions; here, we are in the third one (*ξ* = *l*_B_/*a* = 7.12 Å/2.5 Å = 2.85 >1), where the counterions are confined inside the star and part of them condensed around the arms according to the Manning-Oosawa process. In this case, the simulations yield a counterion radial distribution (ρ(*r*) ~ *r*^2^*c*_i_(*r*)) inside the hydrophilic part, increasing slower than *r*^2^, up to the micelle radius; thus *c*_i_(*r*) is slightly peaked around the center. Molecular dynamics simulations [11,12,13] made for similar *ξ* values (conditions *f* = 10, *N*_a_ = 50 and one charged monomer unit out of 3, *α* = 1/3) show a more peaked *c*_i_(*r*) distribution, both for repeat units and counterions (this one follows the repeat unit distribution).

#### 3.5.3. A Heterogeneous Radial Distribution? Return from Our SANS Experiments

Experimentally, we can only get indirect information about the counterion distribution, using *q*_2_* and assuming that it is linked to an average ionic strength. First, the fact that *q*_2_* is constant below c* suggests a constant inner ionic strength, which would be the case when all, or almost all, counterions are confined (and rather homogenously distributed) inside the star. Second, *q*_2_* has just the same values than for linear PSS at the same nominal concentration *c* ≥ c*; this suggests that the counterion distribution is the same as for linear chains, including Manning-Oosawa condensation around the arms. The observed existence of Manning-Oosawa condensation agrees with findings of simulations [10,11,12,13], but at the same time no effect of center-peaked counterion distribution is shown by *q*_2_*. Observing an average can be linked to the fact that the inner ionic strength is not low enough to yield a sufficiently detectable *l*_p_ increase. Then its value would be irrelevant: the main trend towards chain stretching remains repulsion between all charged repeating units inside the star, even in the strong coupling limit. This agrees also with our finding that *R*/*N*_a_ varies as 1.4 Å: the chain is rather strongly elongated. Above c*, *q*_2_* varies as in linear chain semidilute solutions; we cannot detect any onset of the progressive compaction suggested by simulations in the strong coupling regime [10].

A more extensive discussion would require more information about counterion distribution. This will be studied more precisely, via the variation of the counterion valence in a forthcoming paper.

### 3.6. Final Picture in Real Space

**Figure 6:**
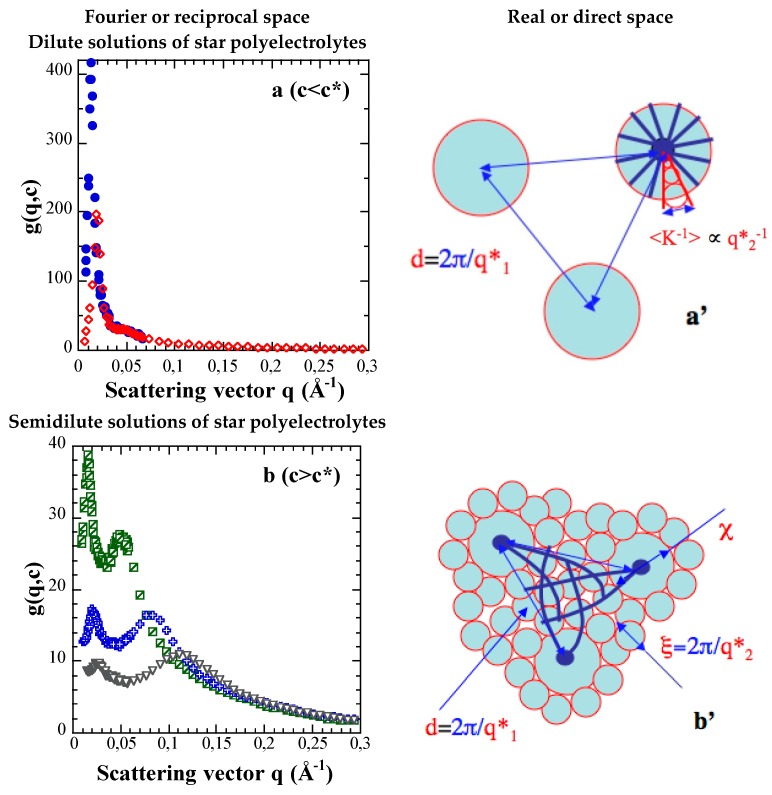
Fourier space (**a**, **b**) *versus* real space (**a’**, **b’**). Relation between the positions of the main maxima of the NaPSS scattering functions in the reciprocal space (*q*_1_* and *q*_2_*) and the mean distances characterizing the average structure of the NaPSS star solutions in the direct space (d, <κ_i_>^−1^, χ and ξ). (**a** and **a’**) S100-S91/D_2_O (*c* < c* ≈ 0.075 mol·L^−1^ – *φ* < φ* ≈ 0.83%); *c* = 0.013 mol·L^−1^ – *φ* = 0.14% (dotted circles); *c* = 0.052 mol·L^−1^ – *φ* = 0.58% (lozenges); (**b** and **b’**) S300-S94/D_2_O (*c* > c* ≈ 0.010 mol·L^−1^ – *φ* < φ* ≈ 0.11%); *c* = 0.088 mol·L^−1^ – *φ* = 0.98% (crossed squares); *c* = 0.230 mol·L^−1^ – *φ* = 2.57% (crosses); *c* = 0.444 mol·L^−1^ – φ = 4.97% (inverted triangles).

Figure 6 shows a schematic picture of the average structures of aqueous solutions of PE stars, linking together their characteristic lengths and the positions of the main maxima of their scattering function. In dilute solution (Figure 6a’), the PE stars are separated from each other, with a liquid-like position order. Their arms are quasi-completely extended. In semi-dilute solution (Figure 6b’), the arms interpenetrate to some extent. The average structure can then be envisioned as a “composite” one made of effective stars of smaller size χ (islands) immersed in a matrix formed by the interpenetrating arm parts, with a constant correlation size *ξ* (sea of blobs). So, the space is partitioned into two media of distinct structure interpenetrated between each other. The effective stars become closer to each other, their size χ is reduced at the benefit of the sea of blobs, as concentration increases. This is one of the two regimes predicted by scaling approaches [14]. We believe that the “shrinking regime” predicted both in [14] and in [10], is not visible. This is supported by the fact that the height of the *q*_1_* peak decreases rapidly, becoming comparable to the height of the *q*_2_* electrostatic peak. The shrinking would correspond to a more intense peak. As said above, more could be obtained from the analysis of the scattering profile, which is beyond the scope of this paper.

Simultaneously, since the arms interpenetrate, the repeat unit concentration *c*_i_ inside the star increases, so both spatial and electrostatic screenings make *ξ* decrease. This results in an increase of *q*_2_*; strikingly, data show that *q*_2_*, for *c* > c*, varies right as the position of the PE peak of the semidilute solutions of linear PSS. This suggests that spatial distributions of both polymer units and counterions are: (i) the same as for linear in average; (ii) rather homogenous (though the peak appears wider than in linear PSS); (iii) the same whatever *N*_a_. These three points suggest that the size χ of the “islands” is limited. The same can be inferred at *c* < c*, from the constancy of *q*_2_* according to *c* (the inner average concentration appears constant), observed for each *N*_a_ (the *q*_2_* value depends on *N*_a_). Therefore the least we can say is that we have no direct evidence of the distribution being peaked on the center of the star.

Finally, let us point out that such an average structure may be encountered only in the present range of functionality, *i.e.*, <*f*> not too little as well as not too large, for highly charged star-branched PEs. It remains that, in this range, it is different from the picture often proposed for neutral stars in good solvent [3,4,52,53,54] where the solvent concentration and the chain average conformation depend on the distance to the center of the star.

## 4. Summary and Conclusions

In summary, complete sulfonation of true PSS arms with convenient functionality (<*f*> = 12) is useful to achieve a simple, geometric picture of the average structure of the aqueous solutions of highly charged hydrophilic polyelectrolyte stars, even simpler than for partially sulfonated stars. This level of simplicity arises from the presence of two maxima in their related scattering functions, one associated with the centers of the stars and another one associated with the arms; the position of each in the reciprocal space having a well-defined and understandable variation with concentration. These variations are differently affected when crossing the critical overlapping concentration c*: above c*, there is no change in the variation function for the center-to-center one (maximum abscissa *q*_1_*) and a drastic one for the branch-to-branch one (maximum abscissa *q*_2_*) that allows defining c*. Remarkably, we found no effect of temperature, confirming the hydrophilic nature of these PSS stars as is nowadays commonly accepted for fully sulfonated PSS.

From the center-to-center average distance 2π/*q*_1_* at *c* = c*, we are able to extract directly a star radius *R*, which varies linearly with the arm degree of polymerization *N*_a_, indicating an extended average conformation for the arms. The front factor of *R* is in agreement with scaling theory and with the wormlike chain model for linear chains. This is in agreement with simulations and theory. On the contrary, the variation of *q*_2_* with *c*, strikingly close to the linear PSS solution case in the semi-dilute regime, does not really evidence the centering effect shown by simulations for the counterion distribution inside the stars. More will be known about the counterion distribution both inside and outside the stars, which will be studied as a function of the counterion valence in a forthcoming paper.

## Figures and Tables

**Table 1:** Characteristics of the PS and NaPSS star macromolecules.

Sample	*X*_L_ %	<*M*_a_>_N_ g·mol*^−^*^1^	*I*_a_	<*N*_a_>_N_	*M*_N_ g·mol*^−^*^1^	*I*	τ_DVB_ %	<*f*>	σ/<*f*>	τ_S_ %	<*m*> g·mol*^−^*^1^	<*v*> cm^3^·mol*^−^*^1^	τ_W_ %	c* mol·L*^−^*^1^	Φ* %
S40	3	3.8 k	1.05	36	40 k	1.13	3.70	10.0	0.35	0	105.1	96.68	0	1.924	18.60
S40-S85	3	–	1.05	36	–	1.13	3.70	10.0	0.35	85 ± 2	188.6	109.90	10.7	0.515	5.66
S100	<2	10 k	1.09	96	126 k	1.06	1.86	10.8	0.32	0	104.6	96.54	0	0.993	9.59
S100-S91	<2	–	1.09	96	–	1.06	1.86	10.8	0.32	91 ± 5	195.8	111.22	10	0.075	0.83
S300	<2	29 k	1.10	280	364 k	1.07	0.64	12.4	0.25	0	104.3	96.45	0	–	–
S300-S94	<2	–	1.10	280	–	1.07	0.64	12.4	0.25	94 ± 5	199.6	111.93	11	0.01	0.11

*X*_L_ is the fraction of residual linear macromolecules; <*M*_a_>_N_ and <*N*_a_>_N_, the number average arm molecular weight and the number average arm degree of polymerization, determined before sulfonation, respectively (<*N*_a_>_N_ = <*Μ*_a_>_N_/m_PS_; m_PS_ is the molar mass of PS monomers (104.15 g·mol^−1^)); *M*_N_, the number average star molecular weight; *I*_a_ and *I*, the arm and star polydispersity indexes, respectively. These characteristics were obtained from SEC measurements on PS arms and PS stars, by using THF as eluent and a low angle laser light scattering spectrometer (LALLS; Chromatix CMX 100) as molecular mass detector (except for S40 arms). <*f*> represents the number average star functionality; σ^2^, the variance of the *f*-distribution; σ, its standard deviation. τ_DVB_ is the DVB content of stars; τ_S_, the arm degree of sulfonation; <*m*> and <*v*>, the molar mass and molar volume of the effective monomers (accounting for the τ_DVD_ and τ_S_ values of the stars [1]), respectively; τ_W_, the weight fraction of water content of dried NaPSS star samples. The overlap concentrations c* associated to PS stars in THFd and NaPSS stars in D_2_O have been estimated as explained in Ref. [1]. For PS stars in D_2_O, it specifically corresponds to the concentration under which *q*_2_* is constant. The overlap volume fraction φ* is then given by the relation φ* = c*<*v*>.

**Table 2:** Characteristics of the NaPSS stars in the dilute regime. <*N*_a_>_N_ is the number average arm degree of polymerization; *R*, the star geometric radius obtained from the position *q*_1_* of the first maximum of the scattering function measured at c*; *q*_2_*, the position of the second maximum of the scattering function measured at *c* ≤ c*.

Sample	<*N*_a_>_N_	*R* (Å)	*q*_2_* (Å^−1^)	*q*_2_*<*N*_a_>_N_ (Å^−1^)	*q*_2_*R
S40-S85	36	53	0.116	4.2	6.1
S100-S91	96	145	0.045	4.3	6.5
S300-S94	280	391	0.0162	4.5	6.3

## Supplementary Materials

Supplementary Materials can be found at www.mdpi.com/2073-4360/8/6/228/s1.

## Abbreviations

The following abbreviations are used in this manuscript:

PE	Polyelectrolyte
SANS	Small Angle Neutron Scattering
SAXS	Small Angle X Rays Scattering
DVB	DiVinylBenzene
PS	Polystyrene
NaPSS	Sodium DiVinylBenzene Polystyrene Sulfonate
SEC	Size Exclusion Chromatography
