# Supplementary material for: SANS from Salt-Free Aqueous Solutions of Hydrophilic and Highly Charged Star-Branched Polyelectrolytes
Source: Polymers (Basel). 2016 Jun 8;8(6):228. doi: 10.3390/polym8060228 (PMC6431935; doi:10.3390/polym8060228)
Supplement: Supplementary file 1 [file polymers-08-00228-s001.pdf]

Supplementary Materials: SANS from Salt-Free Aqueous Solutions of Hydrophilic and Highly Charged Star-Branched Polyelectrolytes

François Boué, Jérôme Combet, Bruno Demé, Martine Heinrich, Jean-Georges Zilliox and Michel Rawiso

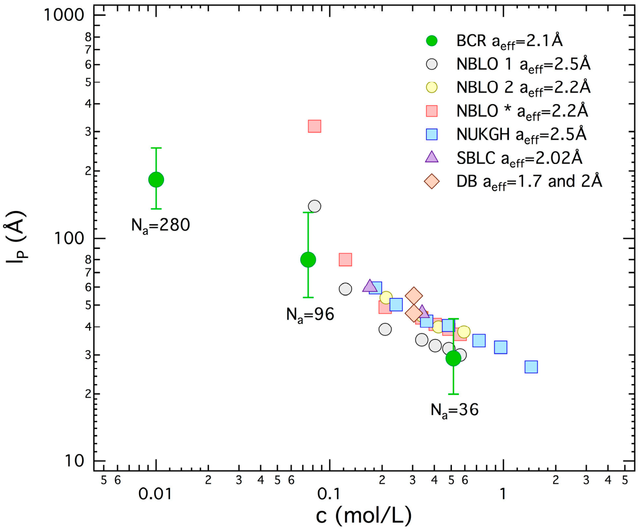

Figure S1. Comparison between the values of l_P obtained from the values of $R = \pi/q_1^*$ at c^* using the wormlike chain model in this paper (noted BCR, Boué-Combet-Rawiso) and the values found in the literature for linear NaPSS solutions. The effective size of the repeat unit a_{eff} is also indicated. NBLO 1 and NBLO 2: Nierlich-Boué-Lapp-Oberthur [1]; NBLO*: Nierlich-Boué-Lapp-Oberthur [2]; NUKGH: Nishida-Urakawa-Kaji-Gabrys-Higgins [3]; SBLC: Spiteri-Boué-Lapp-Cotton [4]; DB: Dubois-Boué [5] (see References below).

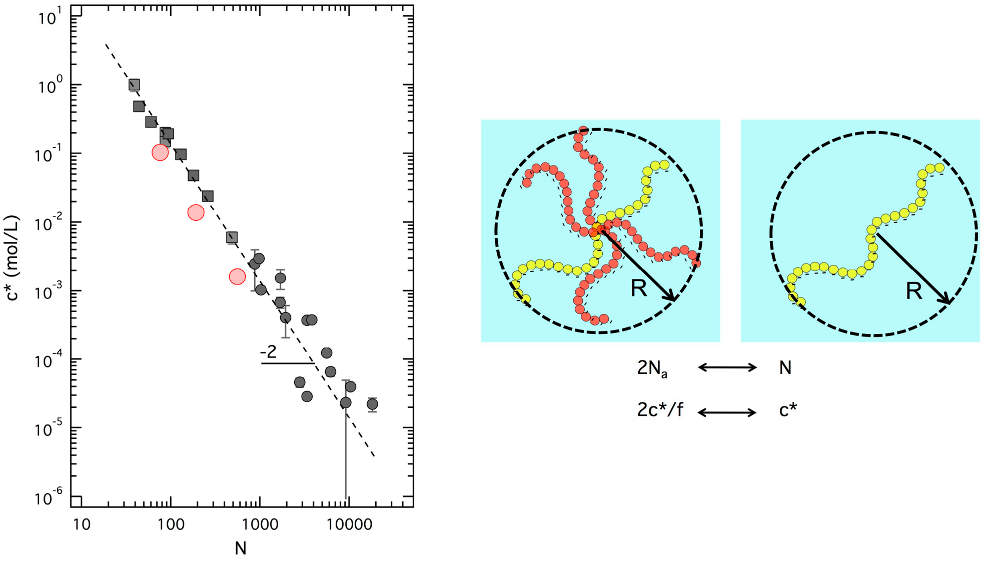

Figure S2. Our three values of c^* (dotted red circles) are plotted versus N (degree of polymerization) on a figure regrouping literature data for different linear NaPSS solutions (Colby *et al.*, Ref. [50] of the paper). Since the architectures are different for linear and star PEs, couples (c^*, N or N_a) cannot be compared directly. To adapt our results to that of linear PEs, we only consider two different arms in the star forming a linear segment of degree of polymerization $2N_a$ (yellow part of the star). The corresponding monomer concentration (and therefore, the equivalent overlap concentration for linear chains) is given by $2c^*/f$.

References

1. Nierlich, M.; Boué, F.; Lapp, A.; Oberthür, R. Radius of gyration of a polyion in salt free polyelectrolyte solutions measured by SANS. *J. Phys.* **1985**, *46*, 649–655.
2. Nierlich, M.; Boué, F.; Lapp, A.; Oberthür, R. Characteristic lengths of the structure of salt free polyelectrolyte solutions. A neutron scattering study. *Colloid Polym. Sci.***1985**, *263*, 955–964.
3. Nishida, K.; Urakawa, H.; Kaji, K.; Gabrys, B.; Higgins, J.S. Electrostatic persistence length of NaPSS polyelectrolytes determined by a zero average contrast technique. *Polymer* **1997**, *38*, 6083–6085.
4. Spiteri, M.N.; Boué, F.; Lapp, A.; Cotton, J.P. Persistence length for a PSS Na Polyion in semidilute solution as a function of the ionic strength. *Phys. Rev. Lett.* **1996**, *77*, 5218–5220.
5. Dubois, E.; Boué, F. Conformation of poly(styrene sulfonate) polyions in the presence of multivalent ions: Small-angle neutron scattering experiments. *Macromolecules* **2001**, *34*, 3684–3697.

© 2016 by the authors; licensee MDPI, Basel, Switzerland. This article is an open access article distributed under the terms and conditions of the Creative Commons Attribution (CC-BY) license (http://creativecommons.org/licenses/by/4.0/).
